# Methodologies Employed in Economic Evaluation of Suicide Prevention Interventions: A Scoping Review

**DOI:** 10.1007/s10488-025-01481-8

**Published:** 2025-12-09

**Authors:** Linda Ryen, Elin Vimefall

**Affiliations:** 1 University Health Care Research Center, Örebro University, Faculty of Medicine and Health, Örebro University, Region Örebro County, Box 1613, 701 16 Örebro, Sweden; 2 Örebro University School of Business, Örebro University, Örebro, Sweden

**Keywords:** Suicide prevention, Economic evaluation, Literature review, Economics of suicide, I19

## Abstract

Economic evaluations can be a powerful tool to assist policymakers in prioritizing interventions to reduce the number of suicides. However, the number of economic evaluations of suicide prevention is low and their quality vary. The aim of this paper is to review the literature on economic evaluations for suicide prevention, specifically focusing on the methods used to measure and value the effect of interventions. To identify articles, we searched PubMed, Web of Science and Scopus for the period from 2000 to 2023. Since the aim was to investigate how a reduction in the number of suicides is measured and valued when evaluating suicide prevention interventions, we only included studies containing effects on mortality. In total 560 unique hits were identified. Most studies were excluded after the first screening of abstracts and titles. The most common reason for exclusion was that the study did not evaluate an intervention for suicide prevention. The final analysis included 16 studies. The number of economic evaluations of suicide prevention is still low, but there is a positive trend and methodological improvements have been made. Nevertheless, several areas where more research is needed were identified, regarding both how to measure and value the effect.

**Supplementary Information:**

The online version contains supplementary material available at 10.1007/s10488-025-01481-8.

## Introduction

Each year, more than 700,000 individuals die by suicide (World Health Organization 2021a). Suicide not only causes great suffering but also leads to significant economic costs, including lost productivity and health care resources. The World Health Organization (WHO) has set a goal of reducing the global suicide rate by one-third by 2030, highlighting the importance of suicide prevention efforts (World Health Organization, 2021b). Economic evaluations can be a powerful tool to assist policy-makers in prioritizing interventions to reduce the number of suicides (McDaid, 2016). However, economic evaluations of suicide prevention have been rare (Feldman et al., 2021; Madsen et al., 2017). One reason might be uncertainty about how to measure and value a reduction in the number of suicides.

To achieve reliable results from economic evaluations on whether interventions are welfare-enhancing or not, it is crucial that both the measurement and valuation of the outcome are accurate. This is not only important for the quality of the specific evaluation but also for understanding how results from different studies can be compared. In order to gain knowledge on this topic, a scoping review was conducted. The aim of this paper is to review the literature on economic evaluations for suicide prevention to investigate the state of the art for economic evaluations of suicide prevention, focusing on which methods are applied for measuring and valuing a reduction in the number of suicides.

Suicide prevention has several characteristics that, in some sense, make it different from other life-saving interventions and that need to be considered when conducting economic evaluations. First, the type of intervention will affect the magnitude of health gain. Preventing suicides by addressing poor mental health implies a higher amount of health is gained than by prevention of the actual suicidal action alone. Second, there might be uncertainty about how much preventing suicides by means restriction reduces the actual suicide rate, whether the suicidal individual will shift to another method or location, or whether the individual will end his or her own life at a later time. Furthermore, depending on whether the individual will receive help, it is unclear what the quality of life of the individual will be post-intervention.

The common feature across all economic evaluation methods is the comparison of costs and consequences following alternative courses of action (Drummond et al., 2015). Costs are measured in the same way regardless of the evaluation method applied, but methods differ in how benefits are measured and valued. In a cost-effectiveness analysis (CEA), the outcome is measured in its natural form, and the result is expressed as the cost per unit of outcome, for example, the cost per suicide averted. In a cost-utility analysis (CUA), the outcome measure includes both the quality and quantity of health gain, for example, by measuring the average amount of quality-adjusted life-years (QALYs) gained. The QALY gain is calculated by multiplying the expected effect on the length of life with the expected health related quality of life (HRQoL) during the saved life years. The HRQoL is measured on a scale between 0 (worst health imaginable) to 1 (perfect health). Hence, one QALY corresponds to one year in perfect health. The result of a CUA is expressed as the cost per QALY gained by the intervention.

In a cost‒benefit analysis (CBA), one instead values both the cost and outcome in monetary terms, for example, by valuing the reduction in suicides with the value of a statistical life (VSL). The VSL is defined as the monetary value of a marginal reduction in the mortality risk that statistically would save one life. This value is often derived either from stated preference studies where respondents are asked about their willingness to pay for a hypothetical good that reduces the mortality risk from a specific cause, or by revealed preference studies where the value for example is derived by analysing wage data to estimate how much individuals demand in higher wage to take a riskier job. In the early days of CBA, the VSL was commonly calculated by estimating the expected effect on productivity, i.e. the loss in production due to the death of one individual. This is called the human capital approach and is not recommended anymore (Andersson and Treich 2011). When all benefits and costs have been valued, the result from a CBA is expressed as the ratio between the monetary value of benefits and costs or as the net present value. Traditionally, the preferred evaluation methods differ depending on the societal sector. Whereas CBA is commonly used in the transportation sector, which is one area for suicide prevention, CUA and, to some extent, CEA are the preferred methods in the health care sector.

Regardless of which method is applied, the basic problem of measuring and valuing the reduction in the number of suicides must be addressed. For CEA, which presents results in terms of the cost per suicide averted, the decision-maker will face a valuation problem when deciding on the intervention. A CUA first requires assumptions of the duration of time in different health states, and then the decision-maker must decide on an acceptable level for the cost per QALY. For CBA, an appropriate shadow value should capture the preferences of the public. In this review, we study which evaluation methods are applied and how the effect from interventions is measured and valued when including a reduction in suicide mortality. Hence, we are addressing the following questions:

- Is the intervention evaluated by CEA, CUA or CBA?
- How is the assumed reduction in suicide mortality measured?
- For CUA and CBA, how is the reduction valued in terms of QALYs gained or shadow price applied?

It does not matter how the outcome is valued if it is not properly measured. Since suicides are rare events, it can often be difficult to obtain enough power to estimate the effect of an intervention. Furthermore, there are different types of interventions that all pose their own challenges for measuring the effect. One approach is to perform an intervention that targets everyone, including those with no or low risk, which is called a *population approach*. The idea is that by reducing the risk for a large group of people, suicides can potentially be prevented even if the initial risk is low. However, these interventions risk being too unspecific, making it difficult to capture an effect. One way to increase specificity without targeting a particular group is by focusing on specific methods for committing suicide. This approach, which is a form of population approach, is known as *means restriction* and involves measures such as installing physical barriers to prevent individuals from using railways as a method of suicide. Even though this often makes it easier to capture the effect of a specific intervention on the suicide rate, it can be difficult to determine whether this reduction translates to a decrease in the overall rate of suicide. Instead of targeting specific methods, an alternative approach is targeting a specific group of people who have been identified to have a high risk of suicide, for example, individuals with previous suicide attempts. This is called the *high-risk approach.* Nevertheless, even when high-risk groups are identified, it is often difficult to obtain enough power to directly capture the effect on the suicide rate from an intervention.

## Materials and Methods

### Search Strategy

This review is based on searches in the electronic bibliographic databases PubMed, Web of Science and Scopus from 2000 to 2023.^1^ The most recent search was performed on March 1, 2024. Potential evaluations were identified by searching abstracts and titles for the following search terms: ‘economic evaluation’, ‘cost benefit’, ‘benefit cost ‘, ‘CBA’, ‘BCA’, ‘cost effectiveness’, ‘CEA’, ‘cost utility’ and ‘CUA’, all in combination with ‘suicide’.

### Eligibility Criteria

The goal was to identify articles containing original economic evaluations of interventions aimed at preventing suicides that were published in peer-reviewed journals between 2000 and 2023 and written in English. The rationale for not including gray literature or older publications is that we aimed to identify the state of the art for economic evaluations performed in a research context.

The first gross list included all articles containing the search terms in the abstract and/or title. All abstracts were read by both authors, who independently judged whether the article should be included. Since the focus of this review is to analyze how a reduction in the number of suicides is measured and valued, only economic evaluations of interventions for which the main goal was to reduce the number of suicides and that specifically incorporated a reduction in mortality were included. Therefore, evaluations that included only suicide attempts without linking this to a reduction in mortality were excluded. Furthermore, we excluded studies where a reduction in the number of suicides was not the main outcome (for example, interventions aiming to reduce the incidence of depression, which in turn could reduce the number of suicides), cost-of-illness studies, review articles, and studies for which no full-length paper was available (i.e., published conference abstracts). All cases yielding different recommendations from the two authors were discussed until a final decision was made.

### Data Extraction Strategy

The identified articles were read in full by both authors and classified by type(s) of evaluation method applied (CEA, CUA, CBA). To perform an overview of the articles, we collected information about the type of intervention, the country of origin and the main result. Then, information more directly connected to our research question was collected. To obtain a better understanding of how effects were measured, this included information about the type of intervention (population approach or high-risk approach), target group, main effect, type of data used to determine the effect size, time perspective and whether a sensitivity analysis was performed. To understand how the effects were valued, the different evaluation methods were then analyzed separately, collecting information relevant for how the valuation is performed for the specific methods.

## Results

### Identified Studies

**Fig. 1:**
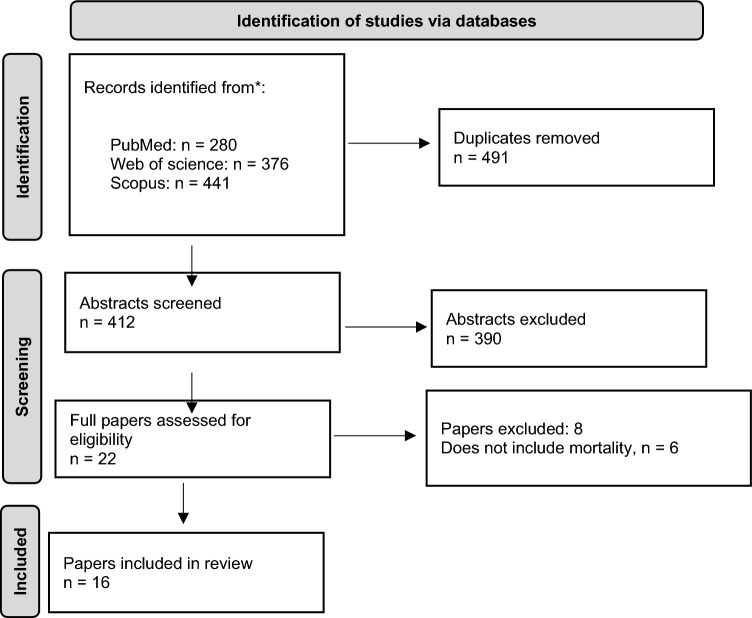
Prisma Flow-diagram, Source: Page et al. (2021)

The final search resulted in 560 unique hits after excluding 491 duplicates. The results from the search are presented in Fig. 1.

Most articles were excluded after the initial screening of the abstracts and titles. The most common reason for exclusion was that the study did not evaluate an intervention for suicide prevention. For some articles, it was not clear from the abstract if the economic evaluation included a reduction in mortality; these were passed on to the next step. After reading the full articles, we excluded 8 more studies since they did not include a reduction in the number of suicides as the main outcome. Sixteen articles were included in the final analysis.^2^

### Characteristics of the Studies

The final sample of included studies is presented in Table 1.

**Table 1:** Characteristics of final sample

Author	Intervention	Country	Evaluation methods	Results
Acolin, (2022)	Dialectical behavioral therapy (DBT) vs cognitive behavioral therapy (CBT) for previous suicide attempters	USA	CUA	DBT is associated with an incremental cost of USD 26,362 per QALY compared to CBT
Bandara et al., (2022)	Barriers at bridge and cliff sites	Australia	1. CBA 2. CEA	1. ROI 2.4 for bridges over 10 years, no significant result for cliff sites 2. ICER: cost saving for bridges, not significant for cliff sites
Bernecker et al., (2020)	Brief cognitive behavioral therapy (BCBT) vs treatment as usual (TAU) for suicidal soldiers	USA	CEA	BCBT is cost saving compared to TAU in the base case scenario
Botchway et al., (2023)	Implementing a suicide prediction tool (OxMIS) for people with severe mental illness	England	CUA	Cost-saving in base-case scenario
Damerow et al., (2020)	Shop-based gate-keeper program for pesticide vendors	Sri Lanka	CEA	At least 0.23 fatal cases needed to be prevented over three years for cost effectiveness
Denchev et al., (2018)	Three emergency department-initiated follow-up interventions (post card, + telephone outreach, + cognitive behavioral therapy (CBT))	USA	CEA	Postcard: cost saving + Telephone outreach USD 4,300 per life year saved + CBT USD18,800 per life year saved
Dunlap et al., (2019)	Screening and intervention post emergency department-visits (ED-SAFE)	USA	CEA	Universal screening is associated with an incremental cost of USD 2,789 per averted suicide attempt or suicide + Intervention USD5,020 per averted suicide attempt or suicide
Flego et al., (2022)	Implementing media guidelines for responsible reporting of suicides	Australia	1. CBA 2. CUA 3. CEA	1. ROI:94 2. Cost saving 3. Cost saving
Hughes et al., (2023)	Prescriptive authority to qualified psychologists	USA	CUA	The incremental net monetary benefit over 20 years was USD 12.81 million per QALY for a WTP of USD 50,000 per QALY. There was > 50% chance of cost-effectiveness for a WTP of USD10 000 per QALY
Kinchin & Doran, (2017)	Workplace suicide prevention program	Australia	CBA	Implementation of the prevention program is associated with a benefit–cost ratio of USD 1.50
Lebenbaum et al., (2020)	Suicide prevention strategy targeting the general population	Canada	CUA	Implementation of the prevention strategy is associated with an incremental cost of CAD18,853 per QALY
Lee et al., (2020)	National bans on highly hazardous pesticides	14 countries	CEA	Low and lower middle-income countries: I$94 per healthy life year gained Upper middle income and high income countries: I$237 per healthy life year gained
Martínez-Alés et al., (2021)	Two strategies for post-discharge suicide interventions compared to treatment as usual: 1. Enchanced contact intervention 2. Psychoterapy program	Spain	CEA	1. Incremental cost is estimated to Euro 2,340 per averted suicide attempt 2. Incremental cost is estimated to Euro 6,260 per averted suicide attempt
Pil et al., (2013)	Suicide helpline	Belgium	CUA	Cost-saving, small health benefits
Vasiliadis et al., (2015)	Nuremberg Alliance against Depression (NAD) multimodel suicide prevention program + 4 community-based suicide prevention strategies	Canada	CEA	The incremental cost associated with implementing the program was CAD 3,979 per life year saved
Whitmer & Woods, (2013)	Suicide barrier on Golden Gate Bridge	USA	CEA	Reported as highly cost-effective. The cost per saved life amounts to USD180,419

Although the total number of articles was low, there was an increasing trend, with zero published between 2000 and 2010, 6 before 2020 and 10 between 2020 and 2023. We defined a study as a CEA if it related the cost to some natural outcome (such as averted suicides), CUA if the outcome used was QALYs^3^ and CBA if the outcome was valued in monetary terms. Some authors state that they perform a return on investment (ROI) analysis but value the outcome with VSL and calculate a benefit–cost ratio. These have been defined as CBA.

Several articles applied more than one method; hence, the total number of evaluations exceed the total number of studies. In the majority of evaluations, CEA (10) was performed; several studies instead conducted CUA (6), and a few used CBA (3). Most studies were conducted in the USA (6), Australia (3), and Canada (2). Almost all studies concluded that the interventions being evaluated had a high probability of being cost-effective.

### Measuring the Effect

To perform a reliable economic evaluation, it is crucial that the outcome is measured in a correct way. However, different types of interventions present different challenges for measuring the effect. In our sample, nine of the studies could be defined as having a population approach, and four of these could be classified as employing means restriction. Furthermore, seven interventions had a high-risk approach (Table 2).

**Table 2:** Measurement of the effect

Author	Type of intervention	Target group	Effect data	Direct effect on	Time perspective	Sensitivity analysis	Comment
Acolin, (2022)	High risk approach	US adults, age 18–64 years with previous suicide attempts	Based on two earlier RCT studies (Brown et al., 2005; McMain et al., 2009)	Suicide attempts and self-harm	1 year	Yes	The model includes the effect from the intervention on attempt. The probability to die from an attempt is then based on Langlois and Morrison (Langlois & Morrison, 2002).
Bandara et al., (2022)	Population approach -Means restriction	Australian public	Based on meta-analysis on 10 studies for bridges and 3 for cliffs	Suicides	5 and 10 years	Yes	Finds no effect on nearby locations in the meta-analysis (included in 10 studies) and does therefore assume no substitution effect
Bernecker et al., (2020)	High risk approach	Suicidal US Army soldiers	Based on an earlier trial (RCT) and data from that trial (Rudd et al., 2015) as well as on data from Army STARRS and CDC WISQARS	Suicide attempts	2 years for treatment effect, lifetime for costs	Yes	Uses historical administrative data to estimate the probability that suicide attempts lead to death in the specific target group
Botchway et al., (2023)	High risk approach	Patience with schizophrenia-spectrum disorder or bipolar disorder in secondary mental health care	Reduction of suicide risk based on Taipale et al. 2021 (Taipale et al., 2021)	Suicides	1 year	Yes	
Damerow et al., (2020)	Population approach—Means restriction	Individuals in rural Sri-Lanka	-	-	3 years	Yes	Perform threshold analysis and do not estimate the effect
Denchev et al., (2018)	High risk approach	Adults presenting to general emergency departments and identified as having a suicide risk	Author opinions and earlier RCT studies, one for each intervention (Brown et al., 2005; Carter et al., 2013; Vaiva et al., 2006)	Suicide attempts or re-attempts	54 weeks	Yes	Assumes that 1/13 attempts results in death based on author opinion in combination with data from American Foundation for Suicide Prevention (2013) and CDC WISQARS
Dunlap et al., (2019)	High risk approach	Patients at emergency departments with suicide ideation or self-harm behavior	Earlier effect evaluation (Miller et al., 2017)	Suicide attempts or suicides	1 year	Yes	Uses a combined measure of suicide attempts and suicides
Flego et al., (2022)	Population approach	Australian public	Earlier meta-analysis for the increased risk of suicides following the reporting on celebrity suicides (Niederkrotenthaler & Sonnek, 2007). Assumes no increase in suicide rates following from responsible media reporting	Suicides	5 years	Yes	
Hughes et al., (2023)	High risk approach	Patients in need of mental health care	CDC WISQARS and a study by Choudhury and Plemmons (Choudhury & Plemmons, 2024)	Suicides and suicide attempts	20 years	Yes	Points out that the assumed effect on the suicide rate was the single most important factor in the sensitivity analysis
Kinchin & Doran, (2017)	Population approach	Australian workers	Earlier study Doran et al. (2016)	Suicides and suicide attempts	1 year	Yes	Assumes that 1/15 attempts lead to death based on WHO statistics
Lebenbaum et al., 2020)	Population approach but including elements of high-risk approach	The public in Ontario, Canada	Earlier studies (Matsubayashi & Ueda, 2011; Hawton et al., 2016)	Suicides and suicide attempts	50 years	Yes	
Lee et al., (2020)	Population approach -Means restriction	Residents in 14 different countries	Earlier systematic review (Gunnell et al., 2017)	Suicides	100 years	Yes	Assumes a gradual decrease in five years which then is supposed to be constant for 100 years. Includes means substitution when calculating the effect on overall suicides
Martínez-Alés et al., (2021)	High risk approach	Adult patients who had attempted suicide	Observational study	Attempt	1 year	Yes	The probability of death per reattempt was obtained from the electronic hospital records of the psychotherapy area
Pil et al., (2013)	Population approach	The public in in Flanders (Belgium)	Earlier study (Gould et al., 2007)	Suicides and suicide attempts	10 years	Yes	
Vasiliadis et al., (2015)	Population approach	Population of Quebec, Canada	Earlier studies (Hegerl et al., 2010; Székely et al., 2013)	Suicides and suicide attempts	1 year	Yes	
Whitmer & Woods, (2013)	Population approach -Means restriction	People in the area	Calculated from the difference in mortality between different means of suicide	Suicides	20 years	-	Assumes that everyone that are prevented to jump from the Golden Gate Bridge will try to end their life with other means. They also assume that 12–13% of the ones that survives an attempt will die from suicide at a later time

Since suicides are rare events, the main problem is achieving enough power to identify an effect directly on the rate of suicides. Several studies (Acolin, 2022; Bernecker et al., 2020; Denchev et al., 2018; Martínez-Alés et al., 2021) specifically among those evaluating high-risk approach interventions, measure the direct effect only on suicide attempts and use data from other sources to estimate the probability of attempts leading to death. Furthermore, one study (Dunlap et al., 2019) used a combined measure of suicide attempts and suicides.

For means restriction interventions, most studies measured the effects directly on the actual number of suicides. It can, however, be difficult to determine whether a reduction in the number of suicides by specific methods corresponds to a reduction in the overall number of suicides or, whether there is a substitution effect for other methods or, in response to different types of physical barriers, for other locations. Among the 4 studies describing means restrictions, three included an estimated effect while one (Damerow et al., 2020) instead calculated the minimal effect needed for the intervention to be cost-effective. Of these three, one assumed a 100% substitution effect (Whitmer & Woods, 2013), one assumed a small substitution effect (Lee et al., 2020), and one assumed no substitution effect at all (Bandara et al., 2022).

A problem related to the substitution effect is determining which assumptions to make about the long-term effect. Preventing a suicide today without treating the underlying problem might mean that the individual tries to commit suicide at a later time, i.e., there can also be a substitution over time. Thereby, not accounting for the future risk of suicide might lead to an overestimation of the effect. The size of this problem depends both on the type of intervention (whether it treats the underlying problem or not), when the suicidal process is interrupted, and on the time horizon for the evaluation. Among the studies included in this review, the time horizon varied from one to one hundred years. Furthermore, 11 out of 16 studies evaluated interventions that, in some broad sense, targeted the mental health or well-being of the individual, which might decrease the probability of future reattempts.

One natural way to handle uncertainty about the size of the effect is to include it in a sensitivity analysis to determine how the assumption affects the result. Sensitivity analyses were performed for almost all (14) of the included studies.

### Valuation

Regardless of the method used for economic evaluation, the basic problem of valuing the outcome must be addressed. The type of evaluation determines which aspects are important, and we will therefore divide the discussion by method. Articles that include several methods are discussed in more than one of the following sections.

#### Cost-Effectiveness Analysis

The method most frequently used for evaluating suicide prevention is CEA, where the cost of the intervention is related to some type of natural outcome (Table 3). By using CEA, the analyst escapes the problem of valuing the outcome of the intervention. However, to determine cost-effectiveness, the cost per outcome needs to be related to some kind of threshold value, if available, or needs to be based on the decision-maker’s willingness to pay for the specific outcome. Another alternative would be to compare the cost-effectiveness of different interventions targeting the same outcome. However, the included studies used somewhat different outcome measures, such as the number of averted suicides (Bandara et al., 2022; Bernecker et al., 2020; Flego et al., 2022; Whitmer & Woods, 2013), life-years saved (Damerow et al., 2020; Denchev et al., 2018; Vasiliadis et al., 2015) healthy life-years gained (Lee et al., 2020) and the combined number of suicide attempts and suicides averted (Dunlap et al., 2019) making such comparisons difficult.

**Table 3:** Cost-effectiveness studies

Author	Main effect	Threshold value referred to
Bandara et al., (2022)	Suicides averted	Cost-saving
Bernecker et al., (2020)	Suicides and suicide attempts averted	Various thresholds, for suicides and suicide attempts, are presented in the sensitivity analysis
Damerow et al., (2020)	Suicides averted	GDP per capita and life year saved used for threshold analysis
Denchev et al., (2018)	Life years saved	Refers to a WTP of USD 50,000 per life-year
Dunlap et al., (2019)	Suicides or suicide attempts averted	Presents the probability of different alternatives being cost-effective depending on WTP for suicides/suicide attempts averted
Flego et al., (2022)	Suicides averted	The intervention is cost saving. Performed threshold analysis reducing the effectiveness of the intervention until it was not cost saving
Lee et al., (2020)	Healthy life years gained (HLYG)	I$100/HLYG is referred to as a threshold value for LMI countries
Martínez-Alés et al., (2021)	Suicide re-attempts averted	Do not use a specific threshold but compares their result to previous results from other studies and recommendations
Vasiliadis et al., (2015)	Suicides averted	Do not use a specific threshold but compares their result to previous results from other studies and recommendations
Whitmer & Woods, (2013)	Suicides averted	VSL used in highway projects

Among the studies that used CEA, all suggested that full or part of the intervention is cost-effective, given one or more specified threshold values. One study (Damerow et al., 2020) referred to GDP per capita as the threshold. Two studies (Denchev et al., 2018; Dunlap et al., 2019) presented either acceptability curves or stated the probability that the intervention was cost effective given different levels of WTP per outcome. Other studies showed that interventions were cost-saving (Lee et al., 2020; Vasiliadis et al., 2015) or related to different VSL values (Bandara et al., 2022).

#### Cost-Utility Analysis

For CUA, the main question is how to estimate the quality of life for a suicidal individual and, in addition, the duration of different health states. Thereafter, the result needs to be related to a threshold value, i.e., the willingness to pay for a QALY, to determine cost-effectiveness. The studies applying CUA are presented in Table 4.

**Table 4:** Cost Utility Analysis

Author	Main effect	Threshold value referred to	Time perspective	Includes re-attempts	Assumptions about quality of life
Acolin, (2022)	QALY	50,000 USD per QALY	1 year	Yes	DALY weights based on expert opinions (Spijker et al., 2011) are used for QALY estimations for untreated suicide ideation and utility weights and utility weights (standard gamble) for mild to moderate depression (Schaffer et al., 2002) are used for treated suicide ideation. For suicide deaths, life years lost with no adjustment for quality of life are used.
Botchway et al., (2023)	QALY	None, but PSA shows 99.96% of iterations are cost-saving	1 year	No	QALY estimations based on utilities for living with severe mental illness (Dilla et al., 2014) and extra reduction for high-risk management (Lenert et al., 2004)
Flego et al., (2022)	QALY	Cost-saving	5 years	No	General population utility scores (Hawthorne et al., 2013) not adjusted for suicide risk
Hughes et al., (2023)	QALY	50,000 USD per QALY referred to in conclusion, but PSA for different WTP thresholds presented	20 years	Yes	Different utilities for those healthy (Fleishman et al., 2005) and for those with a recent non-fatal event (Spijker et al., 2011). Five years in tunnel state after attempt (with increased suicide probability, decreasing for each year (Lebenbaum et al., 2020)
Lebenbaum et al., (2020)	QALY	CAD 50,000 per QALY	50 years	Yes	Adjusted the first year after a suicide attempt according to expert opinion DALY weights (Pil et al., 2013), (Spijker et al., 2011), recover to full utility after one year
Pil et al., (2013)	QALY	Cost-saving	10 years	Yes	Different in different states, lasting one year. Based on expert opinions DALY weights (Spijker et al., 2011) for suicidal thoughts or suicide attempts. For event-free years, a weighted average of general population utility and being suicidal. Utilities adjusted for age

All included studies used QALYs as their main outcome variable. This makes it possible to capture both the effect on length of life or duration of time and on quality of life. Four of the included studies (Acolin, 2022; Hughes et al., 2023; Lebenbaum et al., 2020; Pil et al., 2013) applied an estimate of the effect of being suicidal on quality of life based on the same study by van Spijker et al. (Spijker et al., 2011), in which 16 Dutch medical practitioners assessed the DALY weights for nonfatal suicide attempts. One study instead used the utility score for the general population without adjusting it for an expected decrease in health-related quality of life while being suicidal or after a suicide attempt (Flego et al., 2022). Botchway et al. (Botchway et al., 2023) evaluated two different treatments for patients with severe mental illness using utilities from Dilla et al.(Dilla et al., 2014) for living with such illnesses as well as further reduction from Lenert et al. (Lenert et al., 2004) to account for the disutility following from being treated as a high-risk patient.

However, there is a lack of knowledge about the long-term utility of an individual after a suicide attempt, and four of the studies assumed that the individual would recover to full utility after one year (Hughes et al., 2023; Lebenbaum et al., 2020) or applied a one-year time horizon for the evaluation (Acolin, 2022; Botchway et al., 2023). Pil et al. (Pil et al., 2013) applied a weighted average of the utility of the general population and the utility of being suicidal for those who had had suicidal thoughts during years with no suicide attempt.

#### Cost–Benefit Analysis

Three studies performed a CBA, and their results are presented in Table 5.

**Table 5:** Cost Benefit Analysis

Author	Other costs, savings/benefits included	Method base for VSL	Context for VSL
Bandara et al., (2022)	No	Multiple/WTP	Several
Flego et al., (2022)	Separate analysis with cost savings	Multiple/WTP	Several
Kinchin & Doran, (2017)	Production disturbance, medical cost Administrative costs Other Transfer costs	Human capital approach	Australian workforce

For CBA, the main question is how to value an averted suicide. Two studies used the VSL recommended by the Department of the Prime Minister and Cabinet of Australia: Bandara et al. (Bandara et al., 2022) and Flego et al. (Flego et al., 2022). This value is based on a survey study by Abelson (Abelson, 2012) that included both willingness to pay and revealed preferences studies.^4^ Flego et al. (Flego et al., 2022) used two separate methods to value the reduction in the number of suicides. In addition to using the VSL, they also performed a separate analysis valuing the reduction in suicides by the estimated cost per suicide in Australia. Kinchin and Doran (Kinchin & Doran, 2017) instead used a VSL based on the human capital approach, capturing the loss of potential earnings from the time of death to retirement. In addition to human capital loss, they also included the factors of production disturbance, medical costs, administrative costs, transfer costs and costs for colleagues for counseling and time off.

## Discussion

Economic evaluations are of little use if the benefits of interventions are not reliably measured and valued. For suicide prevention, measuring the effect poses a special challenge and different assumptions made can lead to both overestimation and underestimation of the true effect. First, suicides in general and by specific means in particular are often rare events, making it difficult to achieve enough power to estimate the effects of interventions. Furthermore, the effect on the total number of suicides will depend on substitution effects to other methods, locations and the risk of reattempt in the future.

Among the studies included in this review, the authors treated the potential substitution effect differently. For example, Whitmer and Woods (Whitmer & Woods, 2013) used the conservative assumption that everyone who is hindered from jumping by the suicide barrier on the Golden Gate Bridge will instead use another method to end his or her life. The direct effect of the barrier is therefore only due to the differences in lethality between the different methods. Furthermore, they assumed that among those who survive suicide attempts, 12–13 percent will die from suicide at a later point in time. In contrast, Bandara et al. (Bandara et al., 2022) assumed that none of the individuals who are hindered from jumping will substitute to other methods or commit suicide at a later time. Even though it is likely that the assumptions made in the first study (Whitmer & Woods, 2013) leads to an underestimation of the effect and the other one (Bandara et al., 2022) leads to an overestimation it is difficult to determine which assumption is closest to reality.

Connected to the substitution effect is the question about risk of reattempt. The risk of reattempt has been shown to be highest during the first year following a non-fatal suicide attempt (Bostwick et al., 2016; Christiansen & Frank Jensen, 2007; Demesmaeker et al., 2022). In a meta-analysis of 41 studies, Demesmaeker et al. (Demesmaeker et al., 2022) found that 2.8% of those who had made a non-fatal suicide attempt died by suicide within the first year, 5.6% within five years, and 7.4% within ten years. This can be compared to the incidence of suicide in the population. For example, the age-standardized incidence of suicide in the general population worldwide is estimated to about 9 per 100,000, but there is a large variation between countries (World Health Organization 2021a). These findings indicate that, although the first year is the most critical period, the elevated long-term risk must also be considered. The authors further reported substantial variation across studies, with the risk of reattempt differing by psychiatric diagnosis and age group. Such factors should be considered when making assumptions regarding the duration of the effect and the risk of reattempt. The assumptions made about the substitution effect and risk of reattempt can potentially have large effects on whether interventions are deemed to be cost-effective or not. Ideally, potential substitution effects and reattempts should be measured. However, if not possible we recommend transparency about the assumptions that have been made regarding substitution effects to other methods, locations and points in time. These assumptions should be clearly motivated and tested in a sensitivity analysis, allowing for a nuanced understanding of the risk for over- or underestimation of the effect of an intervention.

A connected problem is the assumptions about quality of life after an averted suicide. The natural way to take this into account is by using a CUA. The QALY concept is commonly used as an outcome measure within the health care sector and allows both effects in terms of loss of life length and loss of quality of life to be considered in the analysis. Unfortunately, there is a lack of knowledge about health-related quality of life postintervention or after a suicide attempt, and several evaluations assumed that an individual returns to the quality of life of the general population after one year, although the time horizons of the studies were longer (Hughes et al., 2023; Lebenbaum et al., 2020). The studies reporting a decreased quality of life while being suicidal or after a suicide attempt (Acolin, 2022; Hughes et al., 2023; Lebenbaum et al., 2020; Pil et al., 2013) all based their QALY estimates on the same study (Spijker et al., 2011). Notably, that study does not present utility weights normally used for calculating QALYs. Instead, it contains expert assessments of disability weights used for calculating DALYs for people with suicidal thoughts and suicide attempts. The QALY and DALY concepts are related, but their differences might lead to systematic variations (Sassi, 2006), and utility weights used for calculating QALYs should be based on individual preferences for different health states. In addition, the included studies do not take long-term quality of life into account. This leaves the question of how long a duration is reasonably needed before it can be assumed that a previously suicidal individual will return to full quality of life. Due to the time component of the QALY concept, the time horizon applied in the studies might affect the result. If it can be assumed that there will be no difference beyond the included time horizon between the general population and those prevented from committing suicide, the results can be considered valid. However, if there is an elevated long-term risk for reattempts or poor health among those who were previously suicidal, there is a risk for results being skewed. As pointed out above, the risk for reattempt varies between different groups and change over the course of time with the highest risk for reattempt during the first year after a non-fatal suicide attempt (Christiansen & Frank Jensen, 2007).

The use of CUA allows a more nuanced picture of the impact of interventions on the analysis and increases the comparability between studies. There is also a prevailing discussion about what threshold values should be applied to establish cost-effectiveness, i.e., what the societal willingness to pay for gaining a QALY would be (Kouakou & Poder, 2022; Ryen & Svensson, 2015) but due to the widespread use of CUA for health care priority-setting, it is possible to compare the cost per QALY to what is commonly accepted when introducing new treatments or interventions.

Ideally, the evaluation should capture both the effect on length and quality of life but more research is needed regarding quality of life and the risk of reattempt after a prevented suicide. Over-simplified assumptions like immediate recovery to full health should be avoided. Furthermore, if no reliable information regarding quality of life is available one should consider if CUA is the right choice of method for the evaluation.

CEA was the most common method for evaluation in this review. Using CEA, the effect is directly compared to the cost in a cost-effectiveness ratio. If several studies use the same outcome, the results from different studies can also be compared to determine which intervention is most cost-effective. Among the studies included in this review, there was large variation in which outcome measures were used. This makes it impossible to compare interventions in terms of cost-effectiveness and to use the studies to answer the question about which intervention prevents suicide at the lowest cost. To increase comparability, we recommend the use of several outcome measures.

For CBA, a reduction in the number of suicides is valued with the VSL.^5^ However, the studies differ in which type of VSL they apply. Some of the studies use a VSL that is based on a human capital approach (e.g., Kinchin and Doran (Kinchin & Doran, 2017)), which at best can be viewed as a lower bound. Today, the standard is instead to use a VSL value based on willingness-to-pay studies, which was also used in two of the included studies (Bandara et al., 2022; Kinchin & Doran, 2017) but it can be questioned whether it is suitable to use the same VSL for suicide prevention as for saving lives in other contexts. It is well known that VSL varies between contexts, and some earlier literature indicates that individuals place less value on reducing the number of suicides than on saving lives from accidents (Covey et al., 2010; Sueki, 2016). However, the results are inconclusive, and Vimefall et al.(Vimefall et al., 2022) did not find any difference between suicide prevention and interventions aimed at reducing the number of deaths from traffic accidents. None of the studies included here used a VSL from the mental health context, and more research is needed to determine whether using the same VSL for suicide prevention as for other interventions is in line with public preferences. The assumptions regarding VSL should always be tested in a sensitivity analysis.

Which evaluation method to be preferred depends on data available and the characteristics of the intervention. If knowing the age profile and future trajectory of health-related quality of life, CUA will allow for capturing the total health gain following from preventing suicides. This health gain can be assumed to differ for interventions aimed at preventing mental ill-health at an earlier stage when compared to interventions preventing the suicidal action as such. Presenting the result as the cost per QALY gained will also allow for interpretation of cost-effectiveness within the health care decision-making framework. On the other hand, if no reliable data on future quality of life is available, instead applying CBA or CEA will in many cases be fairer to policymakers. For interventions performed in settings where CBA is the standard evaluation method applied, like in transport sector, using CBA will allow for policymakers to prioritize between interventions with different aims.

## Limitations

There is a risk that some evaluations are missed due to the limitations of a scoping review. For this topic, evaluations might for example be published as gray literature for suicide interventions performed by public agencies. We do however believe, that for the aim of reviewing the methods used for measuring and valuing the benefits, peer-reviewed publications provide the best picture of the state of the art.

Furthermore, it was not always clear which type of evaluation was performed and in some cases our classification diverges from that of the authors.

## Summary and Conclusion

In this study we have reviewed the literature on economic evaluations for suicide prevention, focusing on the methods used to measure and value the effect of interventions. In total 16 studies were identified. Even if the number of economic evaluations of suicide prevention is still low, there is a positive trend, and methodological improvements have been made. Nevertheless, several areas are identified where more research is needed both regarding how to measure and value the effect.

To measure the effect on the overall number of suicides, there is a need to better understand substitution effects between methods and locations and how these effects differ between interventions. There is also a lack of knowledge about both the short- and long-term health-related utility of an individual after an averted suicide attempt and about the risk of death from suicide at a later time, both of which are crucial for measuring the utility weight to be used in a CUA. Furthermore, to correctly capture the value of a reduction in suicide in a CBA, we need to better understand whether it is in line with public preferences to use the same value of statistical life for suicide prevention as for other interventions that save lives.

## Supplementary Information

Below is the link to the electronic supplementary material.

Supplementary file1

## Footnotes

1 No pre-registered protocol is available for this study.

2 Half of the included studies were funded by some type of government agency. Three were funded by some type of foundation and five stated that no external funding was received or did not state any information regarding funding. None of the included studies were funded by industry.

3 Although QALY is not the only possible outcome measure that can be used for CUA, it was the only generic outcome measure used in the studies included here.

4 Both studies refer to the recommendations from 2014; however, these are no longer available. Since the new guidelines still base their value on Abelson (2007), we assume the same was true for the guidelines from 2014.

5 VSL is also used as the threshold value in some CEAs.
