# Supplementary material for: Methodologies Employed in Economic Evaluation of Suicide Prevention Interventions: A Scoping Review
Source: Adm Policy Ment Health. 2025 Dec 9;53(1):64–76. doi: 10.1007/s10488-025-01481-8 (PMC12823626; doi:10.1007/s10488-025-01481-8)
Supplement: Supplementary file 1 — Supplementary file1 [file 10488_2025_1481_MOESM1_ESM.docx]

# Search example, Web of Science

(TI=(suicide AND economic evaluation)) OR (AB=(suicide AND economic evaluation)) OR (TI=(suicide AND cost effectiveness)) OR (AB=(suicide AND cost effectiveness)) OR (TI=(suicide AND cost utility)) OR (AB=(suicide AND cost utility)) OR (TI=(suicide AND cost benefit)) OR (AB=(suicide AND cost benefit)) OR (TI=(suicide AND benefit cost)) OR (AB=(suicide AND benefit cost)) OR (TI=(suicide AND cea)) OR (AB=(suicide AND cea)) OR (TI=(suicide AND cua)) OR (AB=(suicide AND cua)) OR (TI=(suicide AND cba)) OR (AB=(suicide AND cba)) OR (TI=(suicide AND bca)) OR (AB=(suicide AND bca))

**Filters applied:**

Years:2000-2023,

Language: English,

Document type article
